# Multi-OMICS analyses of frailty and chronic widespread musculoskeletal pain suggest involvement of shared neurological pathways

**DOI:** 10.1097/j.pain.0000000000001364

**Published:** 2018-10-25

**Authors:** Gregory Livshits, Ida Malkin, Ruth C.E. Bowyer, Serena Verdi, Jordana T. Bell, Cristina Menni, Frances M.K. Williams, Claire J. Steves

**Affiliations:** a Department of Anatomy and Anthropology, Sackler Faculty of Medicine, Tel Aviv University, Tel Aviv, Israel; b Department of Twin Research and Genetic Epidemiology, King's College London, London, United Kingdom; c Clinical Age Research Unit, King's College Hospitals Foundation Trust, London, United Kingdom

**Keywords:** Frailty, Chronic pain, Metabolite, GWAS, EWAS, Path analysis

## Abstract

Chronic widespread musculoskeletal pain (CWP) and frailty are prevalent conditions in older people. We have shown previously that interindividual variation in frailty and CWP is genetically determined. We also reported an association of frailty and CWP caused by shared genetic and common environmental factors. The aim of this study was to use omic approaches to identify molecular genetic factors underlying the heritability of frailty and its genetic correlation with CWP. Frailty was quantified through the Rockwood Frailty Index (FI) as a proportion of deficits from 33 binary health deficit questions in 3626 female twins. Common widespread pain was assessed using a screening questionnaire. OMICS analysis included 305 metabolites and whole-genome (>2.5 × 10^6^ SNPs) and epigenome (∼1 × 10^6^ MeDIP-seq regions) assessments performed on fasting blood samples. Using family-based statistical analyses, including path analysis, we examined how FI scores were related to molecular genetic factors and to CWP, taking into account known risk factors such as fat mass and smoking. Frailty Index was significantly correlated with 51 metabolites after correction for multiple testing, with 20 metabolites having *P*-values between 2.1 × 10^−6^ and 4.0 × 10^−16^. Three metabolites (uridine, C-glycosyl tryptophan, and N-acetyl glycine) were statistically independent and thought to exert a direct effect on FI. Epiandrosterone sulphate, previously shown to be highly inversely associated with CWP, was found to exert an indirect influence on FI. Bioinformatics analysis of genome-wide association study and EWAS showed that FI and its covariation with CWP was through genomic regions involved in neurological pathways. Neurological pathway involvement accounts for the associated conditions of aging CWP and FI.

## Abstract 2

Supplemental Digital Content is Available in the Text.

Comparative multiomics of common widespread pain and frailty suggests that shared underlying genetic etiology of these 2 conditions associated with the genomic regions involved in neuroendocrine mechanisms.

## 1. Introduction

Age-related loss of physiological function negatively affects quality of life. This deterioration, defined in general as frailty, leads to an increased risk of illness, dependency, and adverse outcomes, including falls, delirium and disability, and death.^5^ UN reports consistently suggest that populations around the world are aging rapidly (http://www.un.org/en/development/desa/population/publications/pdf/ageing/WPA2015_Report.pdf) and that, between 2015 and 2030, the number of people in the world aged 60 years and older is projected to grow by 56% (from 901 million to 1.4 billion). This situation represents one of the most significant and unprecedented social transformations of the 21st century, with implications for virtually all sectors of society and, in particular, health care systems.

The biological mechanisms underlying frailty have been extensively studied in recent years.^15,26^ We have shown that interindividual variation of frailty scores significantly depends on genetic factors, which explain 34% of their variation. We also found that chronic widespread musculoskeletal pain (CWP)—another prevalent age-related condition—is significantly associated with the Rockwood Frailty Index (FI), and that this association is caused by shared genetic and common environmental factors. Bivariate variance component analysis revealed a strong and significant genetic correlation (*R*_G_ = 0.69 ± 0.02) between the 2 conditions, after adjustment for age, smoking, and body composition (fat mass).

This raises the important question of the nature of the common genetic factors underlying the 2 phenotypes. One approach to the problem is the genome-wide association study (GWAS). Recent history of GWAS, however, shows that although making a significant contribution to understanding the biological architecture of many common complex traits, many studies of small sample size demonstrated results having a very low level of replication, containing extensive false-positive findings, and able to explain only a small proportion of the genetic variance (heritability).^2,24,34^ Modern molecular technologies allow for simultaneous measurement of thousands of measures of biological molecules from DNA polymorphisms (genomics), DNA methylation often causing genes' activation/deactivation (epigenomics), an array of endometabolites (metabolomics), and others. This, in combination with the development of bioinformatics tools, creates the basis for new Multi-OMICS data analysis strategies of a complex phenotype.^23,28^

We have reported previously that CWP is strongly associated with steroid hormone metabolism, in particular with epiandrosterone sulphate (EAS).^12^ Epigenome-wide association study (EWAS) of this sample provided evidence of neurological pathway involvement in CWP.^14^ Thus, the main aim of this study was 2-fold. First, implementing complex genomic, epigenomic, and metabolomic data analysis, we attempted to identify major molecular pathways affecting FI score variation. Second, using comparative statistical and bioinformatics study of the 2 Multi-OMICS outcomes, we attempted to identify common molecular genetic factors involved in the significant genetic correlation between these 2 conditions.

## 2. Materials and methods

### 2.1. Study design

**Figure 1.**
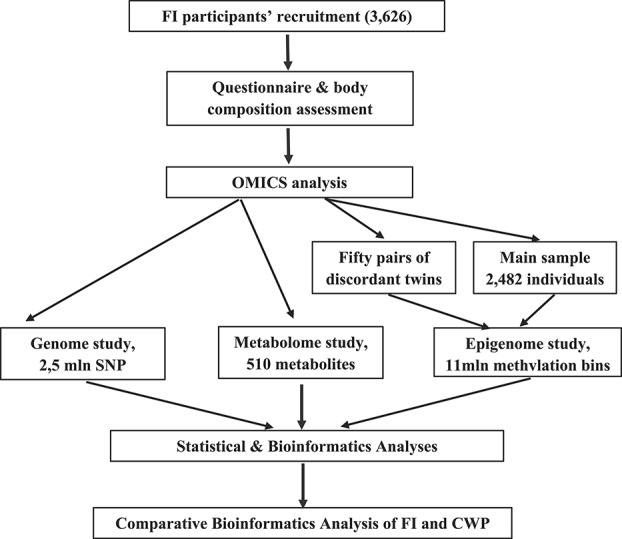
General outline of the study design. CWP, common widespread pain; FI, Frailty Index.

Figure 1 illustrates the major steps undertaken in this study. Further description of the material and methods follows this flow chart.

### 2.2. Sample and study phenotypes

We compared simultaneously metabolomics and genomic factors affecting CWP (assessed by us previously^12,14^) and FI (examined in this article). We described the study sample and the methods of FI assessment in detail elsewhere.^15^ Briefly, participants of this study were 3626 UK female volunteers (with age ranging from 17 to 93 years, with mean 60.5 ± 13.9 years) from the NIHR BRC TwinsUK BioResource. The sample included 1696 monozygotic (MZ) and 1152 dizygotic twins, and 778 singletons ascertained from the general population. Subjects in TwinsUK are sent regular questionnaires for completing and are invited intermittently to attend for a clinical visit. Each participant was assessed for study of primary phenotypes and potential covariates, including age, smoking, basic anthropometric measurements, and body composition as assessed by dual-energy X-ray absorptiometry (DXA) technology. All participants provided written informed consent. The St. Thomas' Hospital research ethics committee approved the project.

Frailty was quantified through the Rockwood FI and was created as a proportion of deficits from 33 binary health deficit domains.^15,22^ Originally, assessment of frailty contained a pain component. In this study, the pain component was omitted, and individual FI scores were recalculated correspondingly, to avoid possible duplication with the CWP assessment. Common widespread pain was assessed using the London Fibromyalgia Epidemiology Study Screening Questionnaire that had been sent to twins for self-completion, without reference to the cotwin,^31^ and has been described by us previously.^12^

### 2.3. Genomics

Genotyping was performed in 3 batches on the Illumina Human Hap300 and Human Hap610-Quad arrays, the results were collated, and quality control was performed. Only single nucleotide polymorphisms (SNP) with genotyping rate ≥95% and nonsignificantly deviating H-W equilibrium, with *P* ≥ 0.0001, were retained in the analysis. The 2.5 mln SNP genotype data were available for 2286 individuals. Details of genotyping and quality control were repeatedly previously reported elsewhere.^32^

### 2.4. Epigenomics

DNA methylation was profiled across the genome using MeDIP-sequencing followed by DNA methylation quantifications to assess epigenome variation, as previously described in these data.^13^ Briefly, the MeDIP-sequencing protocol resulted in an average of 15,684,723 high-quality uniquely mapping reads (Burrows-Wheeler aligner) that were subsequently extended to 350 bp to represent the average MeDIP fragment size. Fragments per kilobase per million were quantified in bins (methylation sites) of 500 bp (250 bp overlap) genome-wide using MEDIPS v1.6.^4^ Methylation levels were finally assessed at 11,524,145 genomic regions of size 500 bp (bins) genome-wide in each individual in the sample (N = 1820). In the epigenome-wide association analysis (EWAS), we only considered bins that displayed significant correlation between longitudinal samples available for each individual, where methylation levels were measured at least 3 years apart; and we denoted these bins as lsBINs.^13^ After quality control, 723,029 lsBINs remained and were considered in the downstream analyses in the study.

### 2.5. Metabolomics

Metabolon Inc performed nontargeted ultrahigh-performance liquid chromatography and mass spectrometry on fasting plasma samples of TwinsUK participants.^27^ Raw data were median-normalized for daily fluctuations of the method and then inverse-normalized. In our sample, 2530 individuals had metabolic traits. Finally, 305 metabolites having complete data in more than 2000 participants were used in this study. However, of them, 103 metabolites represented unknown biochemical compounds and were excluded from the following analyses, despite highly significant correlations in some instances. Further details are given in our previous study.^12^

### 2.6. Statistical analysis and bioinformatics

Statistical analysis was conducted in the following major steps (Fig. 1). First, a series of linear regression analyses was conducted to test for the association between the each of the available metabolites and FI scores, with simultaneous adjustment for age. Metabolites found to be significantly correlated with FI scores, after correction for multiple testing by false discovery rate ([FDR] = 5.0E-04^1^), were selected for further analysis. To establish the independent effect of each metabolite, they were simultaneously examined in multiple regression analysis with FI scores as dependent variable. In addition, we examined the effect of age and relative fat mass (as covariates), taking into account familial structure of the sample using MAN statistical package (http://www.tau.ac.il/∼idak/hid_MAN.htm). Next, implementing GenABLE package (http://www.genabel.org/packages/GenABEL), GWAS of the FI and metabolites significantly associated with it were conducted using the entire sample. Because the analysis discovered a number of significant associations between FI and CWP, we implemented path analysis (http://people.exeter.ac.uk/SEGLea/multvar2/pathanal.html) to determine direction of effects. Further explanation is given below.

An EWAS was conducted. The sample was divided into 2 groups: Gr1, including 50 pairs of frailty discordant MZ twins, and Gr2, including all the remaining individuals (N = 1720). Twin discordance was determined by intrapair difference in FI scores, weighted to pair mean FI score, ie, D_j_ = (FI_j1_ − FI_j2_)/0.5 (FI_j1_ + FI_j2_). The 50 most discordant twin pairs were selected, and methylation levels compared by a paired *t* test. Per bin methylation levels were correlated with FI scores in the Gr2 sample, or compared by paired *t* test between the affected and unaffected on CWP discordant twins and in the remaining sample (N = 1720). *P*-values for the results obtained in 2 nonoverlapping samples were combined using the Fisher method.^8^

Genome-wide association study and EWAS results were subjected to bioinformatic and annotation analyses, for example, implementing gene ontology (GO) methods (Fig. 1). In the epigenome analysis, we first assigned lsBINs to the nearest ENSEMBL gene using *MEDIPS* package for *R*.^11^ For genes with multiple bins assigned, we retained the lsBIN with the lowest *P*-value for association with CWP. Using the Fisher approach, we took the combined *P*-values and conducted GO analysis using the *weight01* algorithm implemented in the *topGO*package for *R* (https://bioconductor.org/packages/release/bioc/html/topGO.html). The statistical significance of overrepresentation of GO terms was estimated using the Fisher exact test. To make the study comparable with our CWP results,^12^ 2 GO domains have been analyzed: biological process and cellular component (CC). QIAGEN's Ingenuity Pathway Analysis (www.qiagen.com/ingenuity) was used for pathway analysis. A similar approach was implemented to summarize and interpret the GWAS data obtained for both FI and CWP.

## 3. Results

### 3.1. Descriptive statistics

Present study characteristics of the phenotypes are given in Table 1 with a detailed description of FI given recently elsewhere.^15^ The preliminary analysis showed that FI scores were significantly correlated with age (*r* = 0.446), body mass index (*r* = 0.326), and relative fat mass, FAT/H^2^ (*r* = 0.330), all having *P* < 0.0001. Frailty Index also strongly depended on smoking habits increasing almost linearly between nonsmokers, previous smokers, and current smokers, respectively: 0.216 (SD = 0.133), 0.242 (SD = 0.138), 0.254 (SD = 0.149), F_(2d.f.)_ = 21.8, *P* = 4.1 × 10^−10^, adjusted for age. In the following analysis, the effect of these covariates was taken into account. Only association with FAT/H^2^ remained significant if tested simultaneously with body mass index.

**Table 1:**
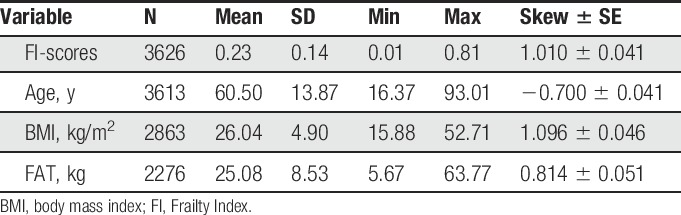
Basic descriptive statistics of the study sample from TwinsUK.

### 3.2. Metabolomics analysis with Frailty Index

There were 305 chemical compounds for analysis after excluding metabolites that had >20% missing values^10^ and with unknown identity. Table 2 shows the 20 most significantly correlated metabolites with FI (*P* = 2.1^−06^ to *P* = 4.0^−16^), which were all significant at *P* < 0.001, after FDR correction for multiple testing.^1^ Variations of these metabolites were not independent and many showed significant intercorrelation with one another. We tested them simultaneously in multivariable regression analysis (Table 3), finding 5 metabolites showing statistically independent effect (*P*-values ranged between 3.6^−03^ and 2.4^−07^). These were EAS, uridine, C-glycosyl tryptophan (C-GT), N-acetyl glycine (N-AG), and indolepropionate. Except C-GT, all metabolite levels decreased with increasing FI scores, especially EAS (*P* = 3.3^−06^). C-GT, indolepropionate, and N-AG are related to protein metabolism, and the first 2 molecules are involved in tryptophan metabolic pathway. However, when the data were examined taking into account familial structure and potential effect of heritability, the results changed slightly (Table 3, 2 right-hand columns), although the general pattern remained the same. The main difference was that indolepropionate was no longer statistically significant.

**Table 2:**
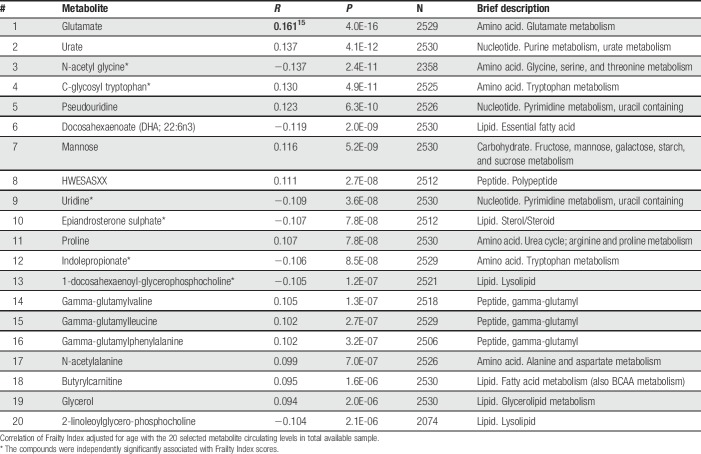
Association of frailty with metabolites.

**Table 3:**
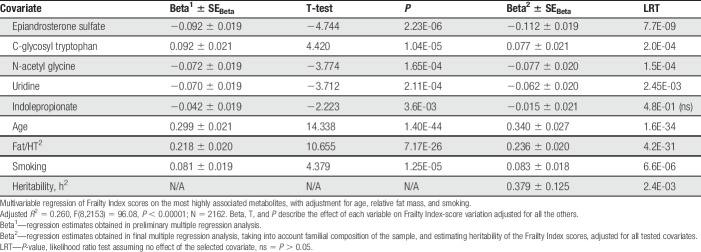
Risk factors for frailty.

We conducted metabolic pathway analysis focusing on pathway enrichment analysis (http://www.metaboanalyst.ca/faces/home.xhtml). This analysis was conducted twice: first, the top 20 metabolites (Table 2) were tested. Next, 51 metabolites with *P*-value ≤0.0002 (corrected for multiple testing) were examined. In the first analysis, galactose, pyrimidine, arginine and proline, and D-glutamine and D-glutamate metabolism pathways were suggested, with *P*-values 0.014 to 0.046. In the second analysis, metabolic pathways of gluconeogenesis, galactose, taurine and hypotaurine, alanine, and some others were suggested, with *P*-values 0.002 to 0.033. After FDR correction, however, no significant metabolic pathways were identified.

### 3.3. Genome-wide association study of metabolites associated with Frailty Index

A multiple linear regression model with additive genetic effect was applied to test for FI score–genotype association using ∼2.5 million genotyped and/or imputed autosomal SNPs. Other covariates adjusted in the model included age and relative fat mass. In addition, we similarly tested each of the metabolites associated with frailty phenotype. The results are presented as series of Manhattan plots (Figure S1 and Table S1, supplementary material 1, available at http://links.lww.com/PAIN/A639). Frailty Index showed no genome-wide significant associations, with top *P*-values ranging between the 10^−3^ and 10^−4^. Genome-wide association study of the 4 significantly associated metabolites showed a different pattern. Three of them, specifically uridine, N-AG, and EAS, displayed strong association with the single genomic region, with the top *P*-values correspondingly: <10^−12^ (Chr#22, mapped to *49304328-49318618bp, rs131794*), <10^−74^ (Chr#2 mapped to *27596107-27584444bp, rs1260326*) and <10^−76^ (Chr#7 mapped to *98994442-99024762bp, rs1581492*). For variation of C-GT, we found no genome-wide significant associations.

### 3.4. Epigenome-wide association study of Frailty Index

Testing 723,029 lsBINs in 50 FI discordant MZ twin pairs (Gr1) implementing paired *t* tests revealed overall N_D_ = 27,485 bins that showed nominally significant associations (*P* < 0.05), and of these, the top 20 association signals were ranged *P* = 7.01^−5^ to 2.17^−6^. Correlation analysis of lsBINs with FI scores in our main sample (Gr2) identified N_M_ = 31,430 nominally significant correlations, with top 20 associations in a range between *P* = 3.76^−5^ and 4.02^−6^. The results of both analyses were combined by the Fisher test, which detected 27,781 nominally significant results showing the same direction of association in both study subsamples. The 20 top combined results are shown in Table S2 (supplementary material 1, available at http://links.lww.com/PAIN/A639). These data, as well as the GWAS data, were subjected to GO analysis (Tables S3–S5, in supplementary material, available at http://links.lww.com/PAIN/A639).

Comparing GO results obtained in GWAS and EWAS, we observed a few common functional genomic regions, defined as “neuron recognition” in biological process category with shared genes: *CNTNAP2, ROBO2*; in CC classification in the category “neuron projection,” the shared genes were *CNTNAP2, ROBO2, CDH13*, and *GRM7*. In addition, in CC classification, the categories “excitatory synapse” and “actin cytoskeleton” were also identified in both GWAS and EWAS analyses. Thus, the genomic/epigenome analyses suggested that the genomic regions associated with functions of nervous system dominate the list of the potential candidate genes.

### 3.5. Comparison of Frailty Index with CWP

Because CWP and FI are highly associated with common shared genetic factors, we were interested whether and to what extent they shared multi-omic characteristics. We have reported the results of the OMICS analyses of the CWP elsewhere.^12,14^ They were compared with the present results. Of 4 metabolites significantly associated with FI score (Table 3), EAS and uridine were also significantly (*P* = 1.05^−9^ and *P* = 5.8^−03^, respectively, after adjustment for covariates) associated with CWP. Comparing the nominal significant results identified in a similar analysis design, we observed 2 potential common pathways: D-glutamine and D-glutamate metabolism and galactose metabolism pathways. However, they were not significant after FDR correction.

Comparing results of GWAS and EWAS implementing GO analysis for both phenotypes, we identified the 2 common groups of genes: (1) “Neuron recognition,” with *P*-values ranging from 2.1^−2^ (EWAS of CWP) to *P* = 3.0^−3^ (GWAS of FI scores), and (2) “Neuron projection (terminus),” with *P*-values ranging from 2.4^−2^ (GWAS of FI scores) to *P* = 1.8^−2^ (EWAS of CWP).

### 3.6. Path analysis of Frailty Index and CWP

**Figure 2.**
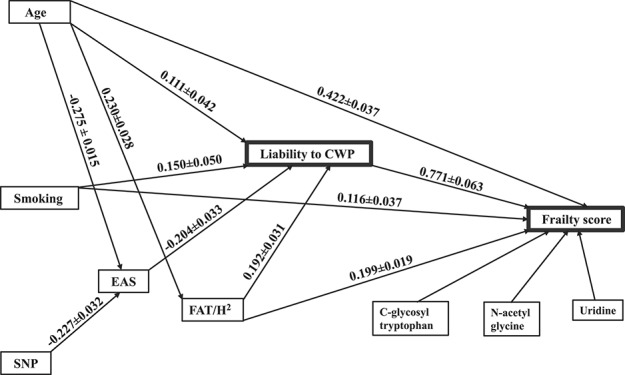
Path analysis of liability to CWP and its potential effect on FI-score variation in relation to several common covariates. The main hypothesis is that CWP manifestation causes FI deterioration. CWP, common widespread pain; EAS, epiandrosterone sulphate; FI, Frailty Index.

We examined a model including the direct and indirect effect of covariates on FI scores through CWP. In other words, we hypothesized that CWP manifestation could be an independent risk factor for worsening FI status of an individual and several studies suggest this sequence of relations between CWP and FI.^29,30^ First, using modified variance decomposition analysis testing the liability-threshold model of dichotomous variables,^16^ we examined the contribution of potential covariates (age, smoking, relative fat mass, EAS levels, and leading SNPs), on CWP. Next, implementing variance decomposition analysis, we estimated all possible direct and indirect effects of CWP manifestation and other covariates on FI scores variation. At this stage, the epigenome signals were not included in the analysis. Figure 2 summarizes the main results of path analysis showing that all tested covariates affect the CWP liability scores significantly. Although age, fat mass, and smoking increase the risk of CWP, EAS circulating levels decrease with raising of the CWP scores.

Evaluating all possible direct and indirect effects on FI scores, we observed that again almost all tested covariates (CWP, age, smoking, and relative fat mass but not EAS levels) exerted a significant effect on FI scores, with clear dominance of the CWP manifestation. Remarkably, when we added C-glycosyl tryptophan, N-acetyl glycine, and uridine to the analysis (identified as independently associated with FI scores (Table 3)), they contributed their independent association to FI (Fig. 2) while not altering other parameter estimates, and their own regression coefficients were virtually the same as reported in Table 3.

## 4. Discussion

As the modern human population is ageing, the prevalence of frailty is increasing. Yet, the specific manifestation of frailty in any individual at a particular age varies tremendously, as does prevalence of frailty among different communities.^25^ It is therefore imperative to clarify the main risk factors for incident frailty as well as its deterioration. Previous studies, including ours, have shown a significant contribution of genetic factors to FI,^6,15,33^ along with other strong risk factors, specifically CWP.^28,29^ Chronic widespread pain in turn has a significant genetic component, which exerts a pleiotropic genetic effect on FI.^15^ The main aim of this study was to clarify the molecular-genetic nature of FI heritability and its correlation with CWP.

OMICS analyses identified 20 top metabolites associated with FI after correction for multiple testing (*P* < 0.0002, Table 2). However, the metabolites themselves are highly correlated and final multiple regression analysis revealed only 4 independently associated metabolites: EAS, C-glycosyl tryptophan, N-acetyl glycine, and uridine (Table 3). Although they represent different facets of human physiology, they seem to be relevant in view of the results obtained in present GWAS and EWAS of this sample, which also suggest involvement of genomic regions associated with the nervous system. Path analysis showed that the latter 3 metabolites were independently associated with frailty, whereas the effect of EAS seemed to be mediated through CWP. Epiandrosterone sulphate circulating levels showed no direct path correlation with FI (Fig. 2), but was highly significantly associated with CWP, which in turn was strongly related to FI.

Epiandrosterone sulphate is a major precursor of testosterone and estradiol and a potential neurosteroid (https://pubchem.ncbi.nlm.nih.gov/compound/epiandrosterone). In addition, EAS is involved in blood pressure regulation (through inhibition of the pentose phosphate pathway) and several other components of blood biochemistry, thus affecting blood circulation in the microvasculature. In our data set, unpublished analysis has also identified this metabolite to be associated with depression and anxiety. A causal role of CWP for FI has been suggested repeatedly in the literature in samples of diverse ethnicity;^29,30^ however, no clear potential mechanism of association was proposed. Our previous studies suggested involvement of neurological pathways in aetiology of CWP,^14^ and showed that its appearance significantly correlates with neuropathic pain features,^20^ and with fatigue and depression.^3,9^ This study further suggests that steroid pathways are involved in the mechanism of interaction between frailty and pain.

The other metabolites that were related to frailty independently of CWP also point to the importance of neuroendocrine mechanisms in frailty. Thus, tryptophan metabolism is critical to the biosynthetic pathway generating serotonin (5-hydroxytryptamine) (Ref. 17; http://themedicalbiochemistrypage.org/nerves.html#5ht), a major neurotransmitter in autonomic nervous system as well as in the CNS. Its function relates to mood, cognition (memory and learning), the regulation of appetite, sleep, and others. In an earlier study from our group, C-gly Trp was also associated with age,^18^ which is correlated with frailty. It is likely that uridine, a component of RNA, may have a synergistic effect with serotonin on brain function by modulating serotonin release.^10^ Some reports have indicated that uridine modulates sleeping patterns, and its administration may affect the course of mental disorder as well as improve memory function and pain.^7^ We have previously found uridine to associate with arterial stiffness in TwinsUK,^19^ and with milk intake.^21^ Also, circulating uridine correlates significantly with the gene-expression levels of the purinergic receptor P2RY2.^19^ N-acetyl glycine also fits the hypothesis of FI worsening association with possible deterioration of nervous system functioning. This enzyme is involved in the degradation of N-acylated proteins, and individuals with N-acetyl glycine deficiency will experience multiple neurological phenomena, eg, convulsions, hearing loss, and difficulty feeding (Human Metabolome www.hmdb.ca/metabolites/HMDB0000532). Thus, all 3 molecules seem to be relevant as potential molecular risk factors for FI development and progression. This conclusion is in agreement with our genomic and epigenome analysis. Although association results observed in both analyses did not reach genome-wide significance, the enrichment analysis of the nominally significant results clearly suggest prevalent association with genomic regions involved in NS functions, such as “neuron recognition,” “neuron projection,” and “excitatory synapse.”

Overall, our data consistently point to the association of neurological pathway markers with progression of FI scores. The association between chronic pain and frailty may be mediated by alterations in sex hormone metabolism.

## Conflict of interest statement

The authors have no conflict of interest to declare.

## Supplementary Material

SUPPLEMENTARY MATERIAL

## Appendix A. Supplemental digital content

Supplemental digital content associated with this article can be found online at http://links.lww.com/PAIN/A639.
