# Supplementary material for: Multi-OMICS analyses of frailty and chronic widespread musculoskeletal pain suggest involvement of shared neurological pathways
Source: Pain. 2018 Oct 25;159(12):2565–72. doi: 10.1097/j.pain.0000000000001364 (PMC6250282; doi:10.1097/j.pain.0000000000001364)
Supplement: SUPPLEMENTARY MATERIAL [file jop-159-2565-s001.docx]

Multi-OMICS Study of Frailty and its Genetic Correlation with Common Widespread Pain. Gregory Livshits, Ida Malkin, Ruth Bowyer, Serena Verdi, Jordana Bell, Cristina Menni, Frances M. K. Williams, Claire J. Steves

Supplementary Material 1. SNPs and bins most highly associated with FI-scores on GWAS and EWAS.

Table S1. Main results of GWAS of FI-scores in total sample (20 most significant terms are presented; * - all definitions are according to http://www.alliancegenome.org/gene/HGNC:36285)

| SNP ID | Chrom | Position | Closest Genes* | Allele 1/2 | MAF | Beta | SE | P-value |
| --- | --- | --- | --- | --- | --- | --- | --- | --- |
| rs4709745 | 6 | 164025974 | LOC107986666 | C/T | 0.303 | -0.140 | 0.032 | 1.4E-05 |
| rs4148005 | 17 | 64394061 | ABCA8 | G/T | 0.309 | 0.136 | 0.032 | 2.3E-05 |
| rs2157592 | 22 | 33849850 | LINC01399 | T/C | 0.171 | 0.169 | 0.040 | 2.4E-05 |
| rs10239543 | 7 | 27267109 | RPL35P4, EVX1 | A/G | 0.121 | -0.194 | 0.047 | 3.0E-05 |
| rs7782529 | 7 | 27264316 | RPL35P4, EVX1 | A/G | 0.121 | -0.194 | 0.047 | 3.1E-05 |
| rs1480008 | 12 | 65384056 | GRIP1 | G/A | 0.408 | -0.126 | 0.030 | 3.4E-05 |
| rs6960464 | 7 | 27266787 | RPL35P4, EVX1 | A/G | 0.121 | -0.193 | 0.047 | 3.4E-05 |
| rs11864855 | 16 | 64647928 | LOC101927676 | A/G | 0.112 | 0.191 | 0.046 | 4.0E-05 |
| rs1265296 | 6 | 4405768 | LOC107986561 | G/A | 0.388 | -0.125 | 0.030 | 4.0E-05 |
| rs6956598 | 7 | 27253321 | EVX1 | C/T | 0.098 | -0.208 | 0.051 | 4.0E-05 |
| rs1732325 | 12 | 115338506 | MIR4472-2, MED13L | T/C | 0.061 | 0.256 | 0.063 | 4.4E-05 |
| rs7991424 | 13 | 45131326 | COX4I1P2, FAM194B | A/G | 0.089 | -0.215 | 0.053 | 4.5E-05 |
| rs6832582 | 4 | 169836208 | PALLD | C/T | 0.147 | -0.171 | 0.043 | 6.2E-05 |
| rs3909525 | 16 | 86161146 | JPH3 ZCCHC14 | A/G | 0.110 | -0.193 | 0.048 | 6.6E-05 |
| rs12670023 | 7 | 7939287 | LOC107986765 | C/A | 0.135 | -0.171 | 0.044 | 8.2E-05 |
| rs734930 | 7 | 69658096 | AUTS2 | T/C | 0.451 | 0.118 | 0.030 | 8.3E-05 |
| rs2625939 | 12 | 93541541 | TMCC3 | T/C | 0.217 | -0.140 | 0.036 | 8.7E-05 |
| rs2041566 | 7 | 7933745 | LOC107986765 | A/G | 0.139 | -0.168 | 0.043 | 9.2E-05 |
| rs258576 | 19 | 19972994 | LOC100421703 | G/A | 0.441 | -0.115 | 0.029 | 1.0E-04 |
| rs10155818 | 7 | 135253628 | LOC101928915, MTPN, LUZP6 | G/A | 0.476 | -0.115 | 0.030 | 1.0E-04 |

Table S2. Twenty best association results obtained by EWAS of FI-scores in two subsamples (50 pairs of discordant twins, and main sample) of UK twins united by Fisher combining probabilities test.

|  | | | |
| --- | --- | --- | --- |
| LsBIN position | | Closest Gene  and distance | P-value |
| Chromosome | Start |  |  |
| 21 | 21768751 | FDPSP6 (-7498) | 4.02E-06 |
| 1 | 106791501 | LOC126987 (-167500) | 6.66E-06 |
| 21 | 37486001 | LOC100133286 (*) | 7.78E-06 |
| 10 | 6664751 | LOC101928150 (*) | 8.62E-06 |
| 12 | 107658501 | SETP7 (6661) | 1.11E-05 |
| 12 | 64728751 | C12orf56 (*) | 1.15E-05 |
| 12 | 128804001 | TMEM132C (*) | 1.15E-05 |
| 5 | 180548501 | OR2V1 (2838) | 1.37E-05 |
| 13 | 40929751 | LINC00598 (*) | 1.45E-05 |
| 9 | 82300001 | TLE4 (*) | 1.98E-05 |
| 17 | 9244751 | STX8 (*) | 2.03E-05 |
| 12 | 128804251 | TMEM132C (*) | 2.41E-05 |
| 7 | 45332501 | LOC100419775 (10718) | 2.43E-05 |
| 3 | 71481751 | FOXP1 (*) | 2.94E-05 |
| 13 | 62689251 | LINC00358 (-85570) | 3.05E-05 |
| 3 | 101278751 | TRMT10C (1929) | 3.38E-05 |
| 1 | 191230001 | HNRNPA1P46 (-113850) | 3.42E-05 |
| 9 | 34975001 | KIAA1045 (*) | 3.43E-05 |
| 7 | 45332251 | LOC100419775 (10968) | 3.51E-05 |
| 6 | 52825501 | GSTA4P (-3181) | 3.76E-05 |

Supplementary Material 2

GO analysis of GWAS and EWAS of FI-scores

Table S3. Main results of GO analysis based on GWAS of FI-scores (20 most significant terms in each domain are presented)

| GO.ID | Term | Annotated | Significant | Expected | Classic |
| --- | --- | --- | --- | --- | --- |
| Biological Process Domain | | | | | |
| GO:0030534 | adult behavior | 28 | 5 | 0.64 | 3.4E-04 |
| GO:0048013 | ephrin receptor signaling pathway | 16 | 4 | 0.37 | 3.6E-04 |
| GO:0007610 | behavior | 105 | 9 | 2.4 | 4.6E-04 |
| GO:0060996 | dendritic spine development | 17 | 4 | 0.39 | 4.6E-04 |
| GO:0061001 | regulation of dendritic spine morphogenesis | 9 | 3 | 0.21 | 8.6E-04 |
| GO:0060997 | dendritic spine morphogenesis | 11 | 3 | 0.25 | 1.6E-03 |
| GO:0097061 | dendritic spine organization | 12 | 3 | 0.27 | 2.1E-03 |
| GO:0050803 | regulation of synapse structure or activity | 42 | 5 | 0.96 | 2.3E-03 |
| GO:0060998 | regulation of dendritic spine development | 13 | 3 | 0.3 | 2.7E-03 |
| GO:0008038 | neuron recognition^1,5^ | 4 | 2 | 0.09 | 3.0E-03 |
| GO:0048814 | regulation of dendrite morphogenesis | 14 | 3 | 0.32 | 3.4E-03 |
| GO:0044708 | single-organism behavior | 71 | 6 | 1.62 | 4.8E-03 |
| GO:0019226 | transmission of nerve impulse | 16 | 3 | 0.37 | 5.1E-03 |
| GO:0051963 | regulation of synapse assembly | 17 | 3 | 0.39 | 6.1E-03 |
| GO:0009987 | cellular process | 1939 | 49 | 44.34 | 7.0E-03 |
| GO:0035176 | social behavior | 6 | 2 | 0.14 | 7.3E-03 |
| GO:0051703 | intraspecies interaction between organisms | 6 | 2 | 0.14 | 7.3E-03 |
| GO:0016358 | dendrite development | 35 | 4 | 0.8 | 7.5E-03 |
| GO:0007169 | transmembrane receptor protein tyrosine kinase signaling pathway | 103 | 7 | 2.36 | 7.8E-03 |

| Molecular Function Domain | | | | | |
| --- | --- | --- | --- | --- | --- |
| GO:0005096 | GTPase activator activity^2^ | 38 | 5 | 0.85 | 1.3E-03 |
| GO:0005070 | SH3/SH2 adaptor activity | 11 | 3 | 0.24 | 1.5E-03 |
| GO:0030695 | GTPase regulator activity | 45 | 5 | 1 | 2.8E-03 |
| GO:0008047 | enzyme activator activity | 69 | 6 | 1.54 | 3.7E-03 |
| GO:0060589 | nucleoside-triphosphatase regulator activity | 50 | 5 | 1.11 | 4.4E-03 |
| GO:0035591 | signaling adaptor activity | 18 | 3 | 0.4 | 6.7E-03 |
| GO:0005080 | protein kinase C binding | 7 | 2 | 0.16 | 9.5E-03 |
| GO:0008066 | glutamate receptor activity | 9 | 2 | 0.2 | 1.6E-02 |
| GO:0005246 | calcium channel regulator activity | 10 | 2 | 0.22 | 2.0E-02 |
| GO:0098772 | molecular function regulator | 220 | 10 | 4.9 | 2.0E-02 |
| GO:0030674 | protein binding, bridging | 27 | 3 | 0.6 | 2.1E-02 |
| GO:0000990 | transcription factor activity, core RNA … | 1 | 1 | 0.02 | 2.2E-02 |
| GO:0000991 | transcription factor activity, core RNA … | 1 | 1 | 0.02 | 2.2E-02 |
| GO:0001640 | adenylate cyclase inhibiting G-protein c… | 1 | 1 | 0.02 | 2.2E-02 |
| GO:0001642 | group III metabotropic glutamate ctiver… | 1 | 1 | 0.02 | 2.2E-02 |
| GO:0004571 | mannosyl-oligosaccharide 1,2-alpha-mannosidase activity | 1 | 1 | 0.02 | 2.2E-02 |
| GO:0008332 | low voltage-gated calcium channel activity | 1 | 1 | 0.02 | 2.2E-02 |
| GO:0010854 | adenylate cyclase regulator activity | 1 | 1 | 0.02 | 2.2E-02 |
| GO:0010855 | adenylate cyclase inhibitor activity | 1 | 1 | 0.02 | 2.2E-02 |
| GO:0015173 | aromatic amino acid transmembrane transporter activity | 1 | 1 | 0.02 | 2.2E-02 |

| Cellular Component Domain | | | | | |
| --- | --- | --- | --- | --- | --- |
| GO:0014069 | postsynaptic density | 35 | 5 | 0.74 | 7.0E-04 |
| GO:0099572 | postsynaptic specialization | 35 | 5 | 0.74 | 7.0E-04 |
| GO:0060076 | excitatory synapse^1^ | 38 | 5 | 0.81 | 1.0E-03 |
| GO:0032589 | neuron projection membrane | 13 | 3 | 0.28 | 2.2E-03 |
| GO:0031256 | leading edge membrane | 31 | 4 | 0.66 | 3.7E-03 |
| GO:0098794 | postsynapse | 78 | 6 | 1.65 | 5.3E-03 |
| GO:0097458 | neuron part | 230 | 11 | 4.88 | 6.9E-03 |
| GO:0098589 | membrane region | 201 | 10 | 4.26 | 7.8E-03 |
| GO:0098590 | plasma membrane region | 170 | 9 | 3.61 | 7.9E-03 |
| GO:0015629 | actin cytoskeleton^2,3^ | 64 | 5 | 1.36 | 1.0E-02 |
| GO:0030673 | Axolemma^5^ | 8 | 2 | 0.17 | 1.1E-02 |
| GO:0031253 | cell projection membrane | 45 | 4 | 0.95 | 1.4E-02 |
| GO:0044463 | cell projection part | 157 | 8 | 3.33 | 1.5E-02 |
| GO:0045211 | postsynaptic membrane | 48 | 4 | 1.02 | 1.8E-02 |
| GO:0016459 | myosin complex | 10 | 2 | 0.21 | 1.8E-02 |
| GO:0044308 | axonal spine | 1 | 1 | 0.02 | 2.1E-02 |
| GO:0097481 | neuronal postsynaptic density | 11 | 2 | 0.23 | 2.1E-02 |
| GO:0043005 | neuron projection^1,5^ | 170 | 8 | 3.61 | 2.4E-02 |
| GO:0030424 | Axon^2^ | 81 | 5 | 1.72 | 2.7E-02 |
| GO:0044456 | synapse part | 122 | 6 | 2.59 | 4.1E-02 |

Table S4. Results of GO analysis of EWAS of 50 pairs of frailty discordant twins (20 most significant terms in in each domain are presented)

| GO.ID | Term | Annotated | Significant | Expected | | classic |
| --- | --- | --- | --- | --- | --- | --- |
| Biological Process Domain | | | | | | |
| GO:1901214 | Regulation of neuron death^1^ | 121 | 13 | 6.9 | | 5.60E-04 |
| GO:0048813 | dendrite morphogenesis^1^ | 58 | 10 | 3.31 | | 1.00E-03 |
| GO:0043547 | positive regulation of GTPase activity^1,5^ | 252 | 27 | 14.38 | | 1.80E-03 |
| GO:0030032 | lamellipodium assembly^3,5^ | 31 | 6 | 1.77 | | 1.90E-03 |
| GO:0006874 | cellular calcium ion homeostasis | 164 | 17 | 9.36 | | 2.60E-03 |
| GO:0032024 | positive regulation of insulin secretion | 37 | 7 | 2.11 | | 2.70E-03 |
| GO:0007270 | neuron-neuron synaptic transmission^1^ | 59 | 10 | 3.37 | | 3.00E-03 |
| GO:0006646 | phosphatidylethanolamine biosynthetic pr… | 6 | 3 | 0.34 | | 3.10E-03 |
| GO:1901379 | regulation of potassium ion transmembran… | 31 | 4 | 1.77 | | 3.10E-03 |
| GO:0018076 | N-terminal peptidyl-lysine acetylation | 2 | 2 | 0.11 | | 3.20E-03 |
| GO:0060125 | negative regulation of growth hormone secretion | 2 | 2 | 0.11 | | 3.20E-03 |
| GO:0000117 | regulation of transcription involved in G2/M transition of mitotic cell cycle ^1^ | 2 | 2 | 0.11 | | 3.20E-03 |
| GO:0048009 | insulin-like growth factor receptor signaling pathway | 16 | 4 | 0.91 | | 8.00E-03 |
| GO:0006893 | Golgi to plasma membrane transport | 24 | 4 | 1.37 | | 8.00E-03 |
| GO:0032012 | regulation of ARF protein signal transduction | 8 | 3 | 0.46 | | 8.00E-03 |
| GO:0035335 | peptidyl-tyrosine dephosphorylation | 42 | 7 | 2.4 | | 8.30E-03 |
| GO:0008038 | neuron recognition ^1,4,5^ | 14 | 4 | 0.8 | | 9.10E-03 |
| GO:0046464 | acylglycerol catabolic process^1^ | 18 | 3 | 1.03 | | 9.10E-03 |
| GO:0002051 | osteoblast fate commitment | 3 | 2 | 0.17 | | 9.10E-03 |
| GO:0061088 | regulation of sequestering of zinc ion | 3 | 2 | 0.17 | | 9.10E-03 |
| Molecular Function Domain | | | | | | |
| GO:0015643 | toxic substance binding^1^ | 8 | 4 | | 0.44 | 4.90E-04 |
| GO:0008237 | metallopeptidase activity | 90 | 12 | | 4.93 | 8.50E-04 |
| GO:0031013 | troponin I binding | 2 | 2 | | 0.11 | 2.90E-03 |
| GO:0047372 | acylglycerol lipase activity^1^ | 3 | 2 | | 0.16 | 8.40E-03 |
| GO:0030618 | transforming growth factor beta receptor, pathway-specific cytoplasmic mediator activity | 3 | 2 | | 0.16 | 8.40E-03 |
| GO:0004468 | lysine N-acetyltransferase activity, acting on acetyl phosphate as donor | 3 | 2 | | 0.16 | 8.40E-03 |
| GO:0004699 | calcium-independent protein kinase C activity | 3 | 2 | | 0.16 | 8.40E-03 |
| GO:1904929 | coreceptor activity involved in Wnt sign… | 3 | 2 | | 0.16 | 8.40E-03 |
| GO:0017160 | Ral GTPase binding^3^ | 3 | 2 | | 0.16 | 8.40E-03 |
| GO:0030676 | Rac guanyl-nucleotide exchange factor activity ^3^ | 10 | 3 | | 0.55 | 1.40E-02 |
| GO:0070006 | metalloaminopeptidase activity | 10 | 3 | | 0.55 | 1.40E-02 |
| GO:0005158 | insulin receptor binding | 10 | 3 | | 0.55 | 1.40E-02 |
| GO:0017124 | SH3 domain binding | 49 | 7 | | 2.69 | 1.50E-02 |
| GO:0001206 | transcriptional repressor activity, RNA polymerase II distal enhancer sequence-specific binding^1^ | 4 | 2 | | 0.22 | 1.60E-02 |
| GO:0008273 | calcium, potassium:sodium antiporter activity | 4 | 2 | | 0.22 | 1.60E-02 |
| GO:0031625 | ubiquitin protein ligase binding | 125 | 13 | | 6.85 | 1.70E-02 |
| GO:0005096 | GTPase activator activity^4^ | 74 | 9 | | 4.06 | 1.80E-02 |
| GO:0008013 | beta-catenin binding^1^ | 29 | 5 | | 1.59 | 1.80E-02 |
| GO:0008331 | high voltage-gated calcium channel activity | 5 | 2 | | 0.27 | 2.60E-02 |
| GO:0004726 | non-membrane spanning protein tyrosine phosphatase activity | 5 | 2 | | 0.27 | 2.60E-02 |

| Cellular Component Domain | | | | | |
| --- | --- | --- | --- | --- | --- |
| GO:0008076 | voltage-gated potassium channel complex^1^ | 31 | 7 | 1.71 | 1.10E-03 |
| GO:0001527 | microfibril | 2 | 2 | 0.11 | 3.00E-03 |
| GO:0031234 | extrinsic component of cytoplasmic side of plasma membrane^1^ | 42 | 6 | 2.32 | 2.20E-02 |
| GO:0045202 | synapse | 303 | 28 | 16.75 | 2.20E-02 |
| GO:0005739 | mitochondrion^3^ | 626 | 40 | 34.6 | 2.30E-02 |
| GO:0031307 | integral component of mitochondrial outer membrane | 5 | 2 | 0.28 | 2.70E-02 |
| GO:0000145 | exocyst | 5 | 2 | 0.28 | 2.70E-02 |
| GO:0035327 | Transcriptionally active chromatin^1,3^ | 5 | 2 | 0.28 | 2.70E-02 |
| GO:0044306 | neuron projection terminus | 43 | 3 | 2.38 | 2.70E-02 |
| GO:0005856 | cytoskeleton | 779 | 63 | 43.06 | 2.70E-02 |
| GO:0030426 | growth cone | 48 | 7 | 2.65 | 2.80E-02 |
| GO:0030659 | cytoplasmic vesicle membrane | 304 | 21 | 16.8 | 2.90E-02 |
| GO:0015629 | actin cytoskeleton^3,4^ | 166 | 16 | 9.17 | 3.20E-02 |
| GO:0032588 | trans-Golgi network membrane | 23 | 4 | 1.27 | 3.40E-02 |
| GO:0014069 | postsynaptic density^5^ | 83 | 9 | 4.59 | 3.60E-02 |
| GO:0005829 | cytosol^4^ | 1827 | 113 | 100.98 | 3.80E-02 |
| GO:0005883 | Neurofilament^1,3^ | 6 | 2 | 0.33 | 3.80E-02 |
| GO:0031932 | TORC2 complex^1^ | 6 | 2 | 0.33 | 3.80E-02 |
| GO:0000930 | gamma-tubulin complex^1,3^ | 6 | 2 | 0.33 | 3.80E-02 |
| GO:0030424 | axon^4^ | 182 | 14 | 10.06 | 4.60E-02 |

Table S5. Results of GO analysis based on EWAS of FI-scores in the main sample of UK twins (20 most significant terms in each domain are presented)

| GO.ID | Term | Annotated | Significant | Expected | Classic |
| --- | --- | --- | --- | --- | --- |
| Biological Process | | | | | |
| GO:0007614 | short-term memory^1^ | 7 | 5 | 0.49 | 2.9E-05 |
| GO:0070588 | calcium ion transmembrane transport^5^ | 113 | 16 | 7.91 | 2.2E-04 |
| GO:0030032 | lamellipodium assembly^2,5^ | 31 | 7 | 2.17 | 6.8E-04 |
| GO:0033555 | multicellular organismal response to stress^4^ | 30 | 5 | 2.1 | 3.0E-03 |
| GO:0006297 | nucleotide-excision repair, DNA gap filling | 10 | 4 | 0.7 | 3.4E-03 |
| GO:0007067 | mitotic nuclear division | 148 | 20 | 10.35 | 4.1E-03 |
| GO:0038028 | insulin receptor signaling pathway via phosphatidylinositol 3-kinase | 2 | 2 | 0.14 | 4.8E-03 |
| GO:0007296 | vitellogenesis | 2 | 2 | 0.14 | 4.8E-03 |
| GO:0060134 | prepulse inhibition | 6 | 3 | 0.42 | 5.6E-03 |
| GO:0035556 | intracellular signal transduction | 1065 | 87 | 74.5 | 6.7E-03 |
| GO:0050890 | cognition | 113 | 15 | 7.91 | 7.0E-03 |
| GO:0000722 | telomere maintenance via recombination | 12 | 4 | 0.84 | 7.2E-03 |
| GO:0007596 | blood coagulation | 145 | 16 | 10.14 | 7.5E-03 |
| GO:0097009 | energy homeostasis | 15 | 4 | 1.05 | 9.3E-03 |
| GO:0010842 | retina layer formation | 7 | 3 | 0.49 | 9.3E-03 |
| GO:2000463 | positive regulation of excitatory postsynaptic potential ^5^ | 13 | 4 | 0.91 | 9.8E-03 |
| GO:0030835 | negative regulation of actin filament depolymerization | 21 | 5 | 1.47 | 1.4E-02 |
| GO:0001935 | endothelial cell proliferation | 53 | 7 | 3.71 | 1.4E-02 |
| GO:0060421 | positive regulation of heart growth | 15 | 4 | 1.05 | 1.4E-02 |
| GO:0045945 | positive regulation of transcription from RNA polymerase III promoter during mitotic cell cycle | 3 | 2 | 0.21 | 1.4E-02 |

| Molecular Function Domain | | | | | |
| --- | --- | --- | --- | --- | --- |
| GO:0005545 | 1-phosphatidylinositol binding^1^ | 9 | 4 | 0.61 | 1.9E-03 |
| GO:0030165 | PDZ domain binding | 37 | 8 | 2.52 | 2.7E-03 |
| GO:0030957 | Tat protein binding | 6 | 3 | 0.41 | 5.2E-03 |
| GO:0000978 | RNA polymerase II proximal promoter sequence-specific DNA binding^1^ | 98 | 14 | 6.66 | 5.5E-03 |
| GO:0005089 | Rho guanyl-nucleotide exchange factor activity^1^ | 48 | 10 | 3.26 | 1.2E-02 |
| GO:0001077 | transcriptional activator activity, RNA polymerase II proximal promoter sequence-specific DNA binding^1^ | 107 | 14 | 7.28 | 1.2E-02 |
| GO:0000987 | core promoter proximal region sequence-specific DNA binding | 101 | 16 | 6.87 | 1.3E-02 |
| GO:0004528 | phosphodiesterase I activity | 3 | 2 | 0.2 | 1.3E-02 |
| GO:0030144 | alpha-1,6-mannosylglycoprotein 6-beta-N-acetylglucosaminyltransferase activity | 3 | 2 | 0.2 | 1.3E-02 |
| GO:0017160 | Ral GTPase binding^2^ | 3 | 2 | 0.2 | 1.3E-02 |
| GO:0042803 | protein homodimerization activity | 324 | 32 | 22.03 | 1.7E-02 |
| GO:0035091 | phosphatidylinositol binding^1^ | 86 | 15 | 5.85 | 2.0E-02 |
| GO:0016247 | channel regulator activity | 56 | 14 | 3.81 | 2.4E-02 |
| GO:0017081 | chloride channel regulator activity | 8 | 3 | 0.54 | 2.5E-02 |
| GO:0035005 | 1-phosphatidylinositol-4-phosphate 3-kinase activity^1^ | 4 | 2 | 0.27 | 2.5E-02 |
| GO:0003779 | actin binding | 144 | 16 | 9.79 | 2.5E-02 |
| GO:0001540 | beta-amyloid binding | 10 | 3 | 0.68 | 2.5E-02 |
| GO:0030676 | Rac guanyl-nucleotide exchange factor activity ^2^ | 10 | 3 | 0.68 | 2.5E-02 |
| GO:0005245 | voltage-gated calcium channel activity | 26 | 7 | 1.77 | 2.8E-02 |
| GO:0004864 | protein phosphatase inhibitor activity | 18 | 4 | 1.22 | 2.9E-02 |

| Cellular Component Domain | | | | | |
| --- | --- | --- | --- | --- | --- |
| GO:0005576 | extracellular region^1^ | 1409 | 72 | 98.38 | 1.3E-03 |
| GO:0005891 | voltage-gated calcium channel complex^5^ | 17 | 6 | 1.19 | 1.8E-03 |
| GO:0000139 | Golgi membrane^1^ | 289 | 31 | 20.18 | 4.7E-03 |
| GO:0005930 | axoneme | 46 | 8 | 3.21 | 1.0E-02 |
| GO:0005739 | mitochondrion^2^ | 626 | 41 | 43.71 | 1.3E-02 |
| GO:0044354 | macropinosome | 3 | 2 | 0.21 | 1.4E-02 |
| GO:0030027 | lamellipodium^5^ | 72 | 10 | 5.03 | 2.5E-02 |
| GO:0031095 | platelet dense tubular network membrane^1^ | 4 | 2 | 0.28 | 2.6E-02 |
| GO:0032591 | dendritic spine membrane | 4 | 2 | 0.28 | 2.6E-02 |
| GO:0005640 | nuclear outer membrane | 10 | 3 | 0.7 | 2.7E-02 |
| GO:0005901 | caveola | 27 | 5 | 1.89 | 3.5E-02 |
| GO:0032433 | filopodium tip | 5 | 2 | 0.35 | 4.1E-02 |
| GO:0005614 | interstitial matrix | 5 | 2 | 0.35 | 4.1E-02 |
| GO:0035327 | transcriptionally active chromatin^1,2^ | 5 | 2 | 0.35 | 4.1E-02 |
| GO:0015629 | actin cytoskeleton^2,4^ | 166 | 19 | 11.59 | 4.3E-02 |
| GO:0030054 | cell junction^1,5^ | 520 | 46 | 36.31 | 4.9E-02 |
| GO:0030286 | dynein complex | 22 | 4 | 1.54 | 5.9E-02 |
| GO:0032045 | guanyl-nucleotide exchange factor complex | 6 | 2 | 0.42 | 5.9E-02 |
| GO:0005883 | neurofilament^1,2^ | 6 | 2 | 0.42 | 5.9E-02 |
| GO:0000930 | gamma-tubulin complex^1,2^ | 6 | 2 | 0.42 | 5.9E-02 |

Table S6. Results of the GO analyses based on EWAS of FI-scores in the main sample and sample of 50 frailty discordant

twins united by Fisher's combined probability test (20 most significant terms in each domain are presented)

| GO.ID | Term | Annotated | Significant | Expected | Classic |
| --- | --- | --- | --- | --- | --- |
| Biological Process Domain | | | | | |
| GO:0007614 | short-term memory^3^ | 7 | 4 | 0.4 | 3.2E-04 |
| GO:1901214 | regulation of neuron death^2^ | 121 | 13 | 6.99 | 6.0E-04 |
| GO:0010765 | positive regulation of sodium ion transport | 14 | 4 | 0.81 | 7.1E-04 |
| GO:0046321 | positive regulation of fatty acid oxidation | 4 | 3 | 0.23 | 7.2E-04 |
| GO:0043547 | positive regulation of GTPase activity^2,5^ | 252 | 28 | 14.57 | 2.2E-03 |
| GO:0035023 | regulation of Rho protein signal transduction^5^ | 66 | 11 | 3.82 | 2.2E-03 |
| GO:0007270 | neuron-neuron synaptic transmission^2^ | 59 | 9 | 3.41 | 3.2E-03 |
| GO:0010632 | regulation of epithelial cell migration | 85 | 9 | 4.91 | 3.2E-03 |
| GO:0055098 | response to low-density lipoprotein part… | 11 | 4 | 0.64 | 3.3E-03 |
| GO:0000117 | regulation of transcription involved in G2/M transition of mitotic cell cycle ^2^ | 2 | 2 | 0.12 | 3.3E-03 |
| GO:0005513 | detection of calcium ion | 6 | 3 | 0.35 | 3.3E-03 |
| GO:0031290 | retinal ganglion cell axon guidance | 12 | 4 | 0.69 | 3.7E-03 |
| GO:0018279 | protein N-linked glycosylation via asparagine | 13 | 4 | 0.75 | 5.0E-03 |
| GO:0007155 | cell adhesion | 662 | 56 | 38.27 | 5.6E-03 |
| GO:0000731 | DNA synthesis involved in DNA repair | 21 | 5 | 1.21 | 5.7E-03 |
| GO:0018105 | peptidyl-serine phosphorylation | 116 | 13 | 6.71 | 6.8E-03 |
| GO:0007420 | brain development | 275 | 25 | 15.9 | 7.1E-03 |
| GO:0048813 | dendrite morphogenesis^2^ | 58 | 8 | 3.35 | 8.7E-03 |
| GO:0008038 | neuron recognition^2,4,5^ | 14 | 5 | 0.81 | 9.4E-03 |
| GO:0046464 | acylglycerol catabolic process | 18 | 4 | 1.04 | 9.4E-03 |

| Molecular Function Domain | | | | | |
| --- | --- | --- | --- | --- | --- |
| GO:0034713 | type I transforming growth factor beta receptor binding | 3 | 3 | 0.17 | 1.7E-04 |
| GO:0005524 | ATP binding | 658 | 57 | 36.6 | 3.0E-04 |
| GO:0035035 | histone acetyltransferase binding | 10 | 4 | 0.56 | 1.5E-03 |
| GO:0005089 | Rho guanyl-nucleotide exchange factor activity ^3^ | 48 | 9 | 2.67 | 4.1E-03 |
| GO:0043015 | gamma-tubulin binding | 8 | 3 | 0.44 | 7.6E-03 |
| GO:0015643 | toxic substance binding^2^ | 8 | 3 | 0.44 | 7.6E-03 |
| GO:0047372 | acylglycerol lipase activity^2^ | 3 | 2 | 0.17 | 8.8E-03 |
| GO:0008046 | axon guidance receptor activity | 3 | 2 | 0.17 | 8.8E-03 |
| GO:0008559 | xenobiotic-transporting ATPase activity | 3 | 2 | 0.17 | 8.8E-03 |
| GO:0005545 | 1-phosphatidylinositol binding^3^ | 9 | 3 | 0.5 | 1.1E-02 |
| GO:0001077 | transcriptional activator activity, RNA polymerase II core promoter proximal region sequence-specific binding^3^ | 107 | 12 | 5.95 | 1.4E-02 |
| GO:0001206 | transcriptional repressor activity, RNA polymerase II distal enhancer sequence-specific binding^2^ | 4 | 2 | 0.22 | 1.7E-02 |
| GO:0035005 | 1-phosphatidylinositol-4-phosphate 3-kinase activity^3^ | 4 | 2 | 0.22 | 1.7E-02 |
| GO:0000978 | RNA polymerase II core promoter proximal region sequence-specific DNA binding^3^ | 98 | 11 | 5.45 | 1.8E-02 |
| GO:0008013 | beta-catenin binding^2^ | 29 | 5 | 1.61 | 2.0E-02 |
| GO:0045296 | cadherin binding | 149 | 15 | 8.29 | 2.0E-02 |
| GO:0015459 | potassium channel regulator activity | 20 | 4 | 1.11 | 2.2E-02 |
| GO:0004674 | protein serine/threonine kinase activity | 216 | 22 | 12.01 | 2.4E-02 |
| GO:0043225 | ATPase-coupled anion transmembrane transporter activity | 5 | 2 | 0.28 | 2.7E-02 |
| GO:0070577 | lysine-acetylated histone binding | 5 | 2 | 0.28 | 2.7E-02 |

| Cellular Component Domain | | | | | |
| --- | --- | --- | --- | --- | --- |
| GO:0000930 | gamma-tubulin complex^2,3^ | 6 | 3 | 0.35 | 3.3E-03 |
| GO:0009925 | basal plasma membrane | 14 | 4 | 0.81 | 6.7E-03 |
| GO:0033017 | sarcoplasmic reticulum membrane | 15 | 4 | 0.87 | 8.8E-03 |
| GO:0005576 | extracellular region^3^ | 1409 | 62 | 81.4 | 1.3E-02 |
| GO:0060076 | excitatory synapse^4^ | 87 | 9 | 5.03 | 1.8E-02 |
| GO:0031095 | platelet dense tubular network membrane^3^ | 4 | 2 | 0.23 | 1.8E-02 |
| GO:0043005 | neuron projection^4,5^ | 382 | 32 | 22.07 | 2.0E-02 |
| GO:0030658 | transport vesicle membrane^3^ | 63 | 7 | 3.64 | 2.5E-02 |
| GO:0031234 | extrinsic component cytoplasmic side of plasma membrane^2^ | 42 | 6 | 2.43 | 2.6E-02 |
| GO:0030054 | cell junction^2,5^ | 520 | 42 | 30.04 | 2.9E-02 |
| GO:0035327 | transcriptionally active chromatin^2,3^ | 5 | 2 | 0.29 | 2.9E-02 |
| GO:0008076 | voltage-gated potassium channel complex^2^ | 31 | 5 | 1.79 | 3.0E-02 |
| GO:0005883 | Neurofilament^2,3^ | 6 | 2 | 0.35 | 4.2E-02 |
| GO:0031932 | TORC2 complex^2^ | 6 | 2 | 0.35 | 4.2E-02 |
| GO:0031258 | lamellipodium membrane | 6 | 2 | 0.35 | 4.2E-02 |
| GO:0000139 | Golgi membrane^3^ | 289 | 26 | 16.7 | 5.0E-02 |
| GO:0005955 | calcineurin complex | 1 | 1 | 0.06 | 5.7E-02 |
| GO:0044326 | dendritic spine neck | 1 | 1 | 0.06 | 5.7E-02 |
| GO:0038038 | G-protein coupled receptor homodimeric complex | 1 | 1 | 0.06 | 5.7E-02 |
| GO:0005663 | DNA replication factor C complex | 1 | 1 | 0.06 | 5.7E-02 |
